# Home Se-Cure: A Home Care Service for Cancer Patients during the COVID-19 Pandemic

**DOI:** 10.3390/ijerph182010913

**Published:** 2021-10-17

**Authors:** Tania Buttiron Webber, Silvia Giuliano, Carlotta Patrone, Irene Maria Briata, Maria Franconeri, Francesca Marceca, Monica Magnani, Fortuna Paciolla, Nicoletta Provinciali, Carlotta Defferrari, Matteo Clavarezza, Mauro D’Amico, Alberto Gozza, Monica Boitano, Mattia Alessio-Mazzola, Isabella Cevasco, Andrea DeCensi

**Affiliations:** 1 Medical Oncology, Galliera Hospital, Mura delle Cappuccine 14, 16128 Genoa, Italy; tania.buttiron@galliera.it (T.B.W.); silvia.giuliano@galliera.it (S.G.); irene.maria.briata@galliera.it (I.M.B.); maria.franconeri@galliera.it (M.F.); francesca.marceca@galliera.it (F.M.); monica.magnani@galliera.it (M.M.); fortuna.paciolla@galliera.it (F.P.); nicoletta.provinciali@galliera.it (N.P.); carlotta.defferrari@galliera.it (C.D.); matteo.clavarezza@galliera.it (M.C.); mauro.d.amico@galliera.it (M.D.); alberto.gozza@galliera.it (A.G.); monica.boitano@galliera.it (M.B.); 2 Office Innovation, Development and Lean Application, Galliera Hospital, Mura delle Cappuccine 14, 16128 Genoa, Italy; carlotta.patrone@galliera.it; 3 Department of Surgical Sciences and Integrated Diagnostics (DISC), University of Genoa, Viale Benedetto XV, 6, 16132 Genoa, Italy; malessiomazzola@gmail.com; 4 Healthcare Professions Structure, Galliera Hospital, Mura delle Cappuccine 14, 16128 Genoa, Italy; isabella.cevasco@galliera.it; 5 European Institute of Oncology (IEO), IRCCS, 20141 Milan, Italy

**Keywords:** COVID-19 pandemic, SARS-CoV-2, cancer patients, home care service, nursing

## Abstract

Cancer patients are exposed to a greater risk of COVID-19 infection, resulting in treatment delays and unnecessary hospitalizations. International authorities have suggested reducing visits to hospitals and guarantee continuity of care. We developed a home care project called Home Se-Cure (HSC) to guarantee the continuity of oral, intramuscular, and subcutaneous cancer therapy during COVID-19. The Home Se-Cure project included cancer patients living near Galliera Hospital. Patients received home visits by registered nurses (RNs), whoperformed blood tests and delivered cancer therapies. Patients were instructed to take drugs after blood test results and therapy confirmation by oncologists. Sixty-six patients decided to participate and 38 declined the service. A customer satisfaction questionnaire was administered to a subgroup of patients participating in the project. The most prevalent disease in the HSC group was prostate cancer. The mean age of the patients in HSC was 78.4 years and 68.9 in the decliner group. The majority of the HSC participants appreciated the project because they could stay at home (71%) and reduce the risk of COVID-19 contagion (67.7%). Compared to decliners, the time the study group saved was 2033 hours. HSC guaranteed the continuity of care during the COVID-19 pandemic by reducing the number of patients in the hospital and avoiding crowds in the waiting room.

## 1. Introduction

In the last year, the severe acute respiratory syndrome coronavirus 2 (SARS-CoV-2) infection has spread worldwide causing the COVID-19 pandemic [1]. COVID-19 infection has affected specific segments of the population with particular severity based on age and comorbidities. In particular, the most vulnerable patients are those with older age and chronic diseases, such as cancer [1,2,3].

Indeed, the immunosuppressed status of cancer patients due to disease or anticancer treatment increases their risk of infection compared with the general population. It was demonstrated that patients with cancer have a higher risk of severe events such as needing mechanical ventilation at ICU admission or dying compared with patients without cancer [4].

This status may expose cancer patients to severe complications from the infection, which may result in treatment delays and unnecessary hospitalizations that could impact disease prognosis and increase costs [5]. Data from a retrospective study conducted in China showed that cancer patients with COVID-19 infection have a higher fatality rate compared with the general population [6].

Moreover, the fear and the risk of contagion cause stress for patients who have to go to hospital for treatment, causing a reduction in therapeutic adherence and a delay in anticancer therapies [7].

Several organizations dealt with cancer patient management during the COVID-19 pandemic with guidelines to reduce visits to hospital, decrease contagion risk [8,9], and guarantee continuity of care [10]. Interruption or delay of chemotherapy is not recommended because a COVID-19 infection may contribute to exacerbate cancer progression [11].

Home care assistance is a service that guarantees a connection between hospital and home. The most common model of home care service was used to support palliative care in patients with severe illness [12], including patients with advanced cancer [13]. Several studies showed a significant reduction of symptom burden and an increase in satisfaction and quality of life (QoL) in patients who received home care support in comparison with patients treated with standard of care [14]. This care model seems to guarantee the continuity of care. So, it may be applied not only in palliative care but also in an emergency setting, as suggested by the Porzio et al. study [15]. Moreover, some studies demonstrated that the home care service impacted favorably on the caregiver’s health [16], whose role was pivotal during the COVID-19 emergency. Indeed, the infectivity rate of COVID-19 seems to be associated with a high risk of contagion of caregivers [17], which may limit the capacity to take care of cancer patients and increase costs for the community. To meet the needs of cancer patients, we implemented the “Home Se-Cure (HSC)” service project in May 2020 to guarantee the continuity of oral, intramuscular, and subcutaneous cancer therapies by delivering treatment at home. Our project aimed at assessing the feasibility of maintaining treatment continuity by a home cancer care service delivering anticancer treatments during the COVID-19 pandemic and at determining the customer satisfaction of such an approach.

## 2. Materials and Methods

The project included patients living in the neighboring areas of the city of Genoa (max. 25 km). The inclusion criteria were: being treated with oral, subcutaneous, and intramuscular therapy and/or requiring blood tests to monitor hematological and biochemical toxicity.

Two different questionnaires were administered: one to the group that participated in the project to assess customer satisfaction and one to the group that declined the project to collect the reason for declination.

Questionnaires were designed specifically for the current project and not previously validated.

Personnel involved were two RNs and one car ambulance driver. There were two home visits per week (Tuesday and Thursday from 8 a.m. to 1 p.m.) for a total of about 15 patients per week. At each visit, the patient signed an informed consent for the blood drawing and the administration of therapy, countersigned by the medical oncologist and the executing nurse.

### 2.1. Home Care Service

Registered nurses and oncologists selected the patients, who were then phoned to schedule an appointment. During the phone call to the patient/caregiver, a telephone triage was done to identify the presence of potential symptoms related to COVID-19 such as fever, cough, dyspnea, diarrhea, anosmia, and dysgeusia. In the presence of symptoms, the patient was invited to come to the hospital to perform a swab. This assessment was performed using a checklist compiled by the RN who performed the telephone triage.

The medical oncologist prescribed the blood tests and/or home cancer therapy to be delivered to the patient. The RN picked up the drugs at the hospital pharmacy and went to the patient’s home equipped with the required personal protective equipment (PPE).

Each patient was issued with a clinical report, signed by the oncologist, indicating the chemotherapy drugs in use and the date of the next appointment. Therapy could only be taken after confirmation by telephone from the RN and the oncologist based on the result of the blood tests, usually in the early afternoon when the drug was delivered. If the blood tests were abnormal, the patient was invited not to take the therapy and to attend the hospital the next day instead for further clinical checks.

The home visit was also done only to perform blood tests in order to monitor hematological toxicities in frail and debilitated patients who would have difficulty coming to the hospital. In these cases, based on the results of the blood tests, a hospital visit was scheduled to ultimately administer supportive therapy (e.g., blood transfusions, nutritional support, and hydration).

The performance status was measured by the Eastern Cooperative Oncology Group–ECOG Scale [18] (Table 1).

The traveling time saved by patients who used the home service and the time lost by the declining group undertaking standard hospital visits were calculated using Google Maps by entering the patient’s address and the hospital address and then duplicated.

Waiting times for the blood test were also assessed and considered based on a previously published analysis [19]. The estimated waiting time for a blood test amounted to 1:46 (hours:minutes). All these times were calculated for patients and caregivers. 

The study was approved by the General Direction and Health Medical Officer of the hospital.

### 2.2. Statistical Analysis

A post hoc power analysis was conducted to calculate the sample size of this pilot study [20]. For a statistical power of 90%, a level of significance of 0.05, and a 10% attrition rate, a total of 65 cases was needed to observe a significant difference in treatment continuity (percentage of theoretical dose divided by the actual dose) of 30% between those who accepted and declined.

Continuous variables were expressed as mean ± standard deviation. Categorical variables were reported as frequencies. The Shapiro–Wilk test was used to identify normally distributed variables. Differences between means were tested with the t-test or the Mann–Whitney *U* test. Categorical variables were tested with the chi-squared test or Fisher’s exact test. A *p*-value of <0.05 was considered statistically significant. Statistical analysis was performed with SPSS (SPSS Inc., Chicago, IL, USA) for Windows.

## 3. Results

Between 1 May 2020 and 31 March 2021, 104 patients were screened for inclusion in the Home Se-Cure project: 66 patients decided to participate, while 38 declined and were considered as the declining group.

Each cycle was typically 21 to 28 days. The main patient characteristics are summarized in Table 2. In particular, most participants in the Home Se-Cure group were males, and most werefemales in the declining group. Participants in the Home Se-Cure group were older with the worst PS compared with patients who declined the service. No differences between groups in comorbidities such as hypertension and diabetes were found. The most common tumor type in the Home Se-Cure group was prostate cancer, and the most common was breast cancer in the declining group.

Forty-four questionnaires were completed: 70.5% by Home Se-Cure patients and 29.5% by patients who declined (Table 3). The main reasons for declining the project were the excuse to leave home and the habit of visiting the Oncology Department.

As shown in Table 4, participants appreciate the Home Se-Cure project in order to avoid contact with COVID-19-infected patients and to be cured while remaining at home. A total of 100% of Home Se-Cure participants stated that they would like to continue the project.

The number of admissions to the Oncology Department of our hospital was 12,480 in 2019 and decreased to 12,150 during 2020, overall a 2.64% decrease. Regarding oncology visits, the number of visits in 2019 was 4531, while in 2020 it was 3861, a decrease of 14.8%. The subsequent investigation was mainly focused on therapy administration, including home treatments. The percentage of patients who underwent chemotherapy cycles in 2020 grew by 4.84% (from 10,075 in 2019 to 10,563 in 2020). Chemotherapy, including home delivery, decreased during the first lockdown period. Specifically, there was a significant decrease in the mean number of chemotherapies from 44.2 ± 7.9 (range: 32 to 64) chemotherapies per day in 2019 to 38.5 ± 7.2 (range: 16 to 54) chemotherapies per day in 2020 (independent samples *t*-test *p* < 0.001). In addition, there was a significant increase in the mean number of chemotherapies from 40.2 ± 7.1 (range: 22 to 64) chemotherapies per day in 2019 to 41.6 ± 9.4 (range: 3 to 68) chemotherapies per day in 2020 (*p* = 0.023).

### Time Saved

An analysis was also conducted on the time saved by patients who used the home-based service and the time spent by the declining group who undertook standard hospital visits (Table 5). Travel time and the waiting time for a blood test result were also added to estimate the effective time to complete the service. The findings indicate that the Home Se-Cure group saved a mean of 30:48 ± 20:14 hours:minutes (range: from 3:48 to 70:00) vs. a loss of 35:44 ± 19:14 (range: from 3:32 to 68:12) hours:minutes per patient in the declining group (*p* < 0.001).

## 4. Discussion

Home Se-Cure is a pilot project aimed at assessing the feasibility of a home care service to maintain treatment continuity by delivering anticancer treatments during the COVID-19 pandemic and to determine the satisfaction and the reasons to decline such an approach.

Ensuring continuity of care for patients, especially those with malignancies, during a pandemic is a tricky aspect of health management. Although several models of home care service have been used to support palliative care in patients with advanced cancer and in an end-of-life setting [13], to our knowledge Home Se-Cure is the first home care service developed to deliver anticancer treatments during the COVID-19 pandemic.

This pilot project has highlighted that the administration of oral, subcutaneous, and intramuscular therapies at home is feasible for cancer patients. We believe our results are generalizable to other medical oncology units given the high proportion of non-endovenous treatments that are now available in the oncology armamentarium.

The results of our customer satisfaction questionnaires highlighted that the Home Se-Cure project had a positive impact on patients during the COVID-19 pandemic as it increased their sense of global care undertaken by health professionals and institutions. In fact, 100% of Home Se-Cure participants stated that they would like to continue the service. Furthermore, our data, which highlighted that 71% of Home Se-Cure participants appreciate the project because they could stay at home and reduce the risk of the COVID-19 contagion, are in line with the results of the survey conducted by Baffert et al., which showed that the changes made in the management of cancer patients during the COVID-19 pandemic were positively received by patients, including avoidance of more than two patients at a time in waiting rooms and introduction of a symptom checklist by telephone the day before therapy [21].

The global quality of life (QoL) of cancer patients during the COVID-19 pandemic has been shown to be significantly lower concerning mainly social, emotional, and cognitive spheres and not physical functioning compared to reference values from cancer patients in stages III-IV obtained under non-epidemic conditions by the European Organization for Research and Treatment of Cancer Quality of Life Questionnaire (EORTC QLQ-C30). This analysis highlighted the importance of QoL, which is distinctly affected during public health emergencies because cancer patients need attention and support from physicians, families, and society [22].

Furthermore, we showed a trend of decreased admissions despite an increase in the treatment delivery cycles in the Oncology Department at our hospital during the pandemic. However, we cannot link the reduction of hospital admissions to the implementation of the Home Se-Cure project. The time saved by patients and their caregivers who avoided distressing travel to the hospital and long waiting times for therapy is an important aspect that could impact the patient’s quality of life and costs, as already reported in an analysis conducted by the University of Alabama [23].

Our pilot feasibility project has several important limitations, including the lack of a quantitative QoL questionnaire and a caregiver burden inventory, two very important outcome measures. One minor limitation of our analysis of the travel time is the calculation using Google Maps, which was based on an estimate depending on the time of the typing (i.e., queues, accidents, traffic, etc.). Although the two patient groups significantly differed in age and performance status, this is not a study limitation in the comparison but is one of our main objectives, namely to assess feasibility and to provide relief by guaranteeing treatment continuity to older and frailer patients. Admittedly, patients enrolled in the Home Secure group were resident closer to the hospital, with a mean difference of 5 hours (30:48 hours/patient in the acceptant group vs. 35:44 hours/patient in the declining group). Unfortunately, our limited resources prevented us from reaching more distant patients who might also be in need of our service. As a matter of fact, our project was supported by the hospital resources and a patient advocacy group with only a few thousand euros, which were added for the transportation of nurses by ambulance.

## 5. Conclusions

Our project is feasible and could guarantee the continuity of the management of cancer patients. The results on the satisfaction of home care provide the background for a larger study focused on quality of life, caregiver burden, and costs. In addition, the project saved a lot of time and displacement for frail patients and caregivers involved in the project. For all of these reasons, we hope that the project can become an innovative patient care model and an integral part of our clinical practice even once the COVID-19 pandemic is over.

## Figures and Tables

**Table 1:** ECOG Scale.

Score	Description
0	Fully active, able to carry out all pre-disease performance without restriction
1	Restricted in physically strenuous activity but ambulatory and able to carry out work of a light or sedentary nature, e.g., light house work, office work
3	Ambulatory and capable of all self-care but unable to carry out any work activities; up and about more than 50% of waking hours
4	Completely disabled; cannot carry out any self-care; totally confined to bed or chair
5	Dead

**Table 2:** Patient characteristics.

Patient Characteristics		Home Se-Cure Group		Declining Group	
		Overall *n* = 66	%	Overall *n* = 38	%
**Gender**	Female	30	45.5	19	50.0
	Male	36	54.5	19	50.0
**Age, mean ± SD**		78.4 ± 8.2		68.9 ± 11.9	
**BMI,** **mean ± SD**		25.4 ± 4.1		26 ± 6.1	
**Comorbidity**	Yes	41	62.1	26	68.4
	No	25	37.9	12	31.6
**PS**	0	43	65.2	33	86.8
	≥1	23	34.8	5	13.2
**Tumor type**	Prostate cancer	28	42.4	5	13.2
	Breast cancer	14	21.2	10	26.3
	Lung cancer	8	12.1	8	21.1
	Colorectal cancer	3	4.5	1	2.6
	Pancreatic cancer	3	4.5	1	2.6
	Other	10	15.2	13	34.2
**Treatment**	Hormone therapy	33	50.0	12	31.6
	Chemotherapy	16	24.2	6	15.8
	Target therapy	6	9.1	11	28.9
	Hormone + target therapy	5	7.6	4	10.5
	Other combo	6	8.5	5	13.1

**Table 3:** Main reasons for declining the Home Se-Cure service.

Answers	%
Habit of visiting the Oncology Department	46.2
Excuse to leave home	46.2
Desire to meet hospital staff	30.8
Desire to see my oncologist during an in-person visit	30.8
Perform different disease tests at hospital	8
Desire to meet other patients	8

Multiple choices questionnaire.

**Table 4:** Home care service customer satisfaction. How did home care service help you?

Answers	%
More comfortable staying at home	71.0
Avoid contagion risk	67.7
Avoid longer waiting time at hospital	51.6
Live alone/no caregiver	41.9
Physical difficulties in moving	25.8
Feel sad and alone	25.8

Multiple choices questionnaire.

**Table 5:** Time (hours:minutes) saved or spent according to the Home Se-Cure group and declining group. Negative values represent time saved and positive values time spent.

Time (Hours)	Home Secure Blood Test Waiting Time (A)	Home Secure Travel Time (B)	Declining Group Blood Test Waiting Time (C)	Declining Group Travel Time (D)	Home Secure Total Time (A + B)	Declining Group Total Time Spent (C + D)
**Mean ± standard deviation**	−22:32 ± 14:21	−8:16 ± 6:39	+26:36 ± 13:40	+9:08 ± 8:30	−30:48 ± 20:14	+35:44 ± 19:14
**Total Time**	−1487:32	−545:32	+1010:32	+347:18	−2033	+1357:54

## Data Availability

Data may be made available for collaborative studies upon reasonable request to the corresponding author.
